# Predictors of well-being, future anxiety, and multiple recurrent health complaints among university students during the COVID-19 pandemic: the role of socioeconomic determinants, sense of coherence, and digital health literacy. An Italian cross-sectional study

**DOI:** 10.3389/fpubh.2023.1210327

**Published:** 2023-09-20

**Authors:** Chiara Lorini, Giuseppe Cavallo, Virginia Vettori, Primo Buscemi, Giulia Ciardi, Patrizio Zanobini, Orkan Okan, Kevin Dadaczynski, Vieri Lastrucci, Guglielmo Bonaccorsi

**Affiliations:** ^1^Department of Health Science, University of Florence, Florence, Italy; ^2^Health Literacy Laboratory, Department of Health Science, University of Florence, Florence, Italy; ^3^School of Specialization in Public Health, University of Florence, Florence, Italy; ^4^Department of Sport and Health Sciences, Technical University Munich, Uptown München-Campus D, Munich, Germany; ^5^Department of Health Science, Fulda University of Applied Sciences, Fulda, Germany; ^6^Center for Applied Health Sciences, Leuphana Universitat Lüneburg, Lüneburg, Germany; ^7^Epidemiology Unit, Meyer Children’s University Hospital, Florence, Italy

**Keywords:** psychological determinants, well-being, socioeconomic determinants, university students, SOC, cross-sectional study

## Abstract

The pandemic deeply changed young adults’ life. Lockdown period and the social restrictions dramatically affected university students’ mental health. The aim of our cross-sectional study was to describe psychological well-being, future anxiety (FA), and health complaints (HCs) in a sample of 3,001 students of the University of Florence in the middle of the first two pandemic waves. We assessed the role of subjective social status, chronic diseases, sense of coherence (SoC), and digital health literacy (DHL) as predictors of psychological well-being, FA, and HCs. Students expressed high levels of FA and reported being disturbed by not being able to achieve their desired future goals. About 40% reported a low or a very low well-being and 19.1% experienced two or more subjective health complaints more than once a week. The likelihood of having a better mental health status significantly increased with increasing SoC and among males. Subjective Social Status proved to be a predictor for FA. Enhancing SoC could improve the health status of the university students during the pandemic and beyond.

## Introduction

Italy was the first European country to be severely affected by a high incidence of COVID-19 cases (1, 2). During the first wave, from 1st March to 31 May 2020, Italy registered 227,972 cases and 34,079 deaths (3). All non-essential services and activities (including schools and universities) were suspended, and a “stay-at-home” order was imposed. The pandemic has profoundly changed everyone’s lives and young adults in particularly have suffered severe consequences because they have been unable to maintain social contacts and activities during the crucial phase of their lives. In this context, people with chronic diseases or physical impairments could have been more affected, due to their more vulnerability.

Like many other Universities worldwide, the University of Florence – the setting of the present study - rapidly adapted its organizational processes by adopting distance learning, library closures, virtual dissertation, and a strict application of anti-contagion measures. Recent literature shows that the lockdown period and the other social restrictions dramatically affected university students’ mental health (4). Many studies conducted worldwide presented consistent results showing increasing prevalence of suicidal thoughts, severe distress, perceived stress, depressive symptoms, anxiety, concentration disorders, and psycho-somatization (5–11).

This could be considered because of the syndemic – that is the biological and social interactions between conditions and states, interactions that increase a person’s susceptibility to harm or worsen their health outcomes. In fact, a combined effect of the pandemic and the infodemic has been observed, where the damages caused by SARS-CoV-2 are to be attributed not only to the direct effects on health, but also to the plethora of information produced, part of which unintentionally – misinformation - or deliberately - disinformation – false. In addition to the impact on mental health, Savage et al. (4) observed an increase in sedentary behavior and, in line with another research (12), a reduction in time spent with and level of energy expenditure due to physical activity, especially in males. In summary, although the pandemic was handled differently around the world and the epidemic situation differed across countries, some common patterns in the way the pandemic has impacted health outcomes and behaviors in university students have emerged in the literature.

Nonetheless, to the best of our knowledge, to date just a few studies have investigated the predictors of well-being, anxiety and health complains among university students during the COVID-19 pandemic, although such information would be useful to manage similar situations that may arise again in the future. Since the infodemic increased the state of uncertainty and fear (13) and negatively affected the strategies adopted to contain the contagion, the role of health literacy (HL), particularly of digital health literacy (DHL), has become crucial. DHL has been defined as a set of skills needed by the individuals to search health information on the Internet, and to understand and apply them to increase awareness and responsibility for one’s own health (14). The Internet and the social networks are often used by university students as a primary source of health information (15, 16). However, they frequently have problems in finding the appropriate information on a particular health-related topic and difficulties in assessing the quality and reliability of the information found (17, 18). Moreover, people with chronic diseases seem to be less satisfied with information during the infodemic than those without such health problems, and they pay much more attention to information about COVID-19 because of their higher risks (18).

Another factor that supports individuals in coping with difficulties while maintaining a good physical and mental quality of life is the sense of coherence (SoC). SoC is an important concept of the Salutogenesis theory and according to Antonovsk (19), SoC reflects a persons’ ability and coping capability to respond to stressful situations (20). In this sense, it is a global orientation to see life as structured, manageable, and meaningful (20). On this topic, Leung et al. (21) conducted a study with older adults during the COVID-19 pandemic that showed how SoC directly affected anxiety. Moreover, in this cross-sectional study, the level of DHL was closely related to that of SoC, suggesting that promoting HL, especially its digital component, may be key to improving SoC. Regarding university students, Chu et al. (22) examined the level of SoC and found a positive association with health awareness. and negative links with higher levels of stress and poor financial status (22).

The aim of our study is to describe psychological well-being, future anxiety, and health complaints (HCs) in a sample of students enrolled at the University of Florence, between the first and the second pandemic wave of COVID-19, and to assess the role of subjective social status, chronic diseases, SoC and DHL as their predictors.

## Materials and methods

Study participation was voluntary. Data was collected using an online questionnaire developed by Dadaczynski et al. (17). The Italian version of the questionnaire was developed using a standard procedure of translation and back-translation (23) and includes scales or parts of scales already validated at the national or international level to collect data regarding sociodemographic condition, life situation, future anxiety, DHL and information seeking behaviors, personal health situation (17). The materials and methods are reported in Table 1.

**Table 1:** Materials and methods used for this study.

Sections	Description
Population	51,883 students of University of Florence attending all study courses (bachelor, master, PhD, Postgraduate School) in the academic year 2019/2020.
Respondents and study size	A total of 3,001 (5.8%) students filled in the questionnaire (convenience sample).
Study design	Cross-sectional study conducted in accordance with the Helsinki declaration and approved by the Ethics Committee of the University of Florence (n. 108, 2020/07/07).
Study info	Conducted as part of a large-scale international university students survey (more than 70 countries) launched within the COVID-HL research network (24).
Inclusion criteria	Attending a course at the University of Florence.
Exclusion criteria	None.
Data management	In accordance with the European Regulation 2016/679 and the Legislative Decree 101/2018, all the data have been processed anonymously and cannot be attributed to a specific person.
Data collection	Data were collected between 17th August 2020 and 3rd October 2020, corresponding to the timeframe between the first and second wave of the COVID-19 pandemic in Italy.
Bias	The online questionnaire was sent to all the students through the institutional email; two reminder emails were sent between 17th August 2020 and 3rd October 2020.
*Questionnaire sections*	
Sociodemographic information	Gender, age, and country of origin were collected.
The subjective social status (SSS)	Measured using the MacArthur Scale (25, 26): the participants were asked to place themselves on a ladder with 10 steps (from 1 to 10) where the highest (score of 10) reflects people who have greater economic resources, a prestigious job, and a high level of education. Socioeconomic status was investigated by also asking “How sufficient do you consider the money at your disposal?,” with the following response options: (1) not sufficient, (2) less sufficient, (3) sufficient, (4) completely sufficient (27).
SoC	Measured by means of the nine-item SoC instrument, which explores the following three dimensions: “comprehensibility” (four items), “manageability” (two items) and “meaningfulness” (three items) (28, 29). While the original tool is focusing on work-life exclusively (30) we adapted the introductory question to life in general. It includes nine bipolar adjectives (items) that could be rated on a seven-point (from 1 to 7) semantic differential scale. A SoC scale score was calculated as the mean value of the item scores (31). In our study, it presented a good internal consistency (Cronbach’s alpha: 0.874).
DHL	Assessed by using five of the seven subscales of the Digital Health Literacy Instrument (DHLI), which has been adapted to the COVID-19 pandemic situation (17, 32). The subscales included comprise the following sub-dimensions of the DHLI: (1) searching for information on the web on COVID-19; (2) adding self-generated content on COVID-19; (3) evaluating the reliability of COVID-19-related information; (4) determining personal relevance of COVID-19-related information; (5) protecting privacy on the internet. Each subscale is composed of three items to be answered on a 4-point scale (from 1 “very difficult” to 4 “very easy”; except for “protecting privacy” subscale: from 1 “never” to 4 “often”). According to Lorini et al. (33), a total scale score of DHLI was calculated as the mean value of the single items, excluding cases with more than 5 missing and the “protecting privacy” subscale, due to many criticalities presented in the validation study (low Cronbach’s alfa, high percentage of missing values, better results in the factor analysis when excluding that subscale).
Psychological well-being	Investigated using the WHO-5 scale, a 5-item scale developed by World Health Organization. WHO-5 is composed of five statements regarding (health-related) feelings over the previous 2 weeks, with a six-point response option describing their frequency (0 = at no time; 1 = some of the time; 2 = less than half of the time; 3 = more than half of the time; 4 = most of the time; 5 = all the time). A total score was calculated as the sum of the score for each item, multiplied by 4. The total score ranged from 0 (worst wellbeing) to 100 (best wellbeing) and was grouped into three possible categories: very low well-being (0–30), low well-being (31–50), and high well-being (> 50) (34, 35). It presented an excellent good internal consistency (Cronbach’s alpha: 0.904).
Future anxiety (FA)	To assess students’ negative attitude towards the future. It is a nine-items tool with Likert-type response options; the first five refer to a short version of the future anxiety scale (Dark Future Scale), while the last four belong to the long form of the future anxiety scale (8, 36). For each item, the response options were from 0 (decidedly false) to 6 (decidedly true). In our study, it presented a, acceptable internal consistency (Cronbach’s alpha: 0.718).
Health complaints (HC)	Investigated by eight items, originally included into the health behavior in school-aged children (HBSC) study questionnaire. They addressed how often the students had presented eight complaints in the last 6 months, with five responses options (0 = rarely; 1 = about every month; 2 = about every week; 3 = more than once a week; 4 = about every day) (37–39). Students reporting two or more HC more often than once a week or about everyday were classified as students with “multiple recurrent health complaints.”
Chronic disease and of impairment by health problems	Presence/absence.
*Statistical analysis*	
Distribution	Normality of continuous variables was assessed using the Kolmogorov–Smirnov test.
Descriptive	Continuous variables were described using mean and standard deviation (SD), or median and interquartile range (IQR) as appropriate. Categorical variables were presented as percentages.
Associations	To assess associations between mental health (well-being, future anxiety, and multiple recurrent health complaints) and the continuous variables (age, SoC score and DHLI score), Student’s *t*-test, ANOVA or Kruskal–Wallis test for independent samples were performed, as appropriate, while Fisher’s exact test was applied for categorical variables (sex, SSS, satisfaction with financial situation, presence of chronic disease and of impairment by health problems).
Multivariate logistic regressions	Three different models of multivariate logistic regressions (A, B and C) were performed, one for each variable related to the health mental status: - in model A, the outcome variable was psychological well-being (“high well-being” vs. “low plus very low well-being”) - in model B, the outcome variable was the future anxiety (FA score < 3.67 vs. ≥ 3.67, where 3.67 is the median value of collected data) - in model C, the outcome variable was multiple health complaints (< 2 vs. ≥ 2). In each model, all the variables significantly associated with each outcome variable at the univariate analysis were included as independent variables. Then, using the backward stepwise procedure, independent variables not significantly associated with the outcome were removed. The final models contained only the independent variable significantly associated with the outcome.
Type error	For all the analyses, an alpha level of 0.05 (type I error) was considered as significant.
Software	The analyses were conducted using IBM SPSS 27.0 and Stata 17/SE StataCorp LLC.

## Results

### Description of the sample

A total of *N* = 3,001 undergraduate students participated in the study. The majority was pursuing a bachelor’s degree (62%), while 37% were in a master’s degree programe and a small percentage (1%) were in other courses (PhD, Post-graduate School). Twenty-three percent were enrolled in Humanities and Education degree, 15% in Human Health Sciences, 13% in Engineering, 11% in Mathematical, Physical and Natural Sciences, 10% in Architecture, and another 10% in Economics. The remaining 18% were enrolled in Political Science, Law, Agriculture, and Psychology. About 68% (67.9%) were female and 92.5% were born in Italy. The median age was 22 (IQR: 20–24, range: 18–70 years). About 14% of the subjects (*N* = 408; 13.6%) reported a low SSS, 69% (*N* = 2,072) a medium SSS, and 17.4% (*N* = 521) a high SSS. Moreover, 18.9% were completely sufficient with the money at their disposal, 50.8% sufficient, 24.3% less than sufficient, and 6% not sufficient. The median SoC score was 3.67 (IQR: 2.89–4.44). The median of the total DHLI score was 2.83 (IQR: 2.58–3.17). At least one chronic disease affected 14.7% (*N* = 440) of the respondents and 8.7% (*N* = 262) had functional limitations in daily activities.

### Well-being, future anxiety, and health complaints: general findings

**Figure 1:**
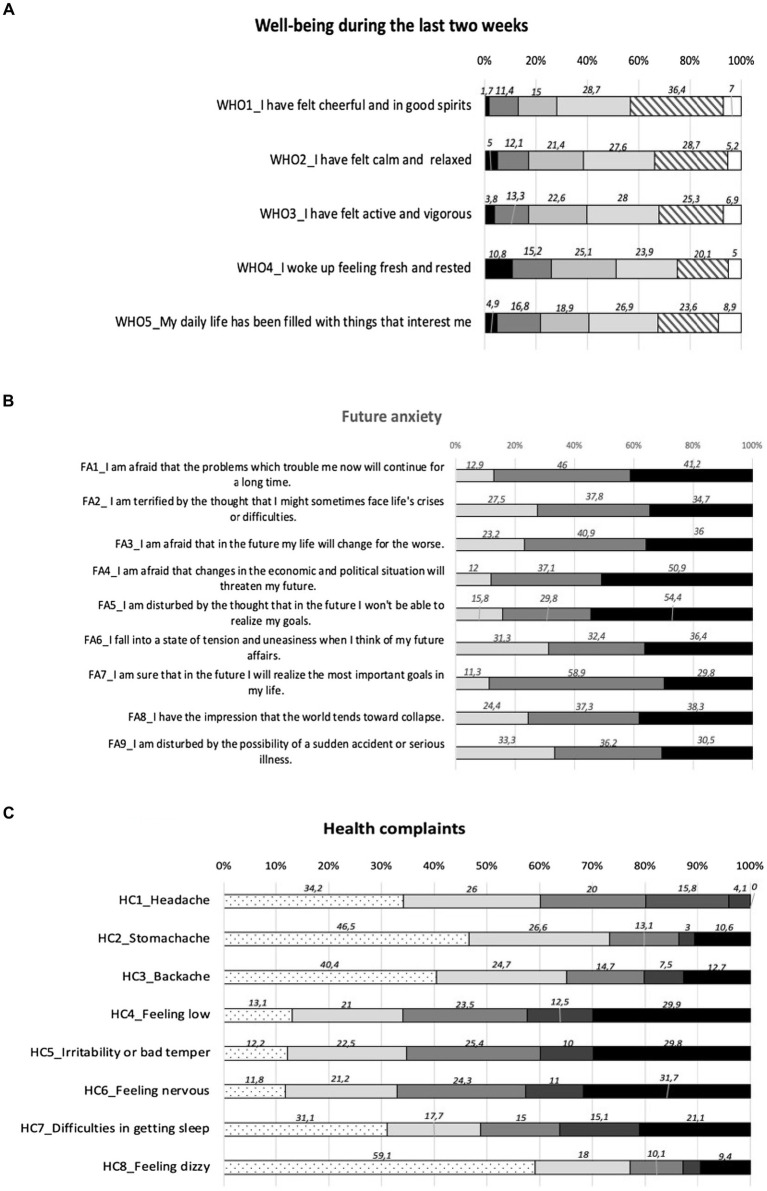
**(A)** Well-being: items responses. 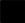 At no time; 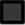 some of the time; 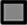 less than half of the time; 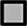 more than half of the time; 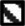 most of the time; 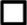 all of the time. **(B)** Future anxiety: items responses. 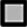 Decidedly false; 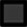 hard of say; 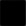 decidedly true. **(C)** Health complaints: items responses. 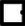 Rarely or never; 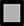 about every month; 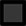 about every week; 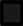 more than once a week; 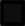 about every day; 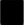 missing.

Concerning the results derived from the WHO-5 scale, 60.8% of the respondents had a high well-being, while 24.5% showed a low well-being and 14.7% a very low well-being. In particular, the highest criticality was observed for the item 4 (“I woke up feeling fresh and rested”), with 10.8% responding with “at no time” and 15.2% with “some of the time” (Figure 1A). The median score at the WHO-5 scale was 56 (IQR: 40–72), with a mean of 55.23 ± 21.9. Future anxiety were mainly related to the possibility that changes in the economic and political situation would threaten students’ future (50.9% of “decidedly true” response) and by the thought that in the future they would not be able to realize their goals (54.4% of “decidedly true” response; Figure 1B). The median score at the FA scale was 3.67 (IQR: 2.67–4.33), while the mean 3.53 ± 1.24. Concerning health complaints, 19.9% reported headache about every day or more than once a week, 12.5% feeling low about every day, and 15% difficulties in getting sleep about every day (Figure 1C). Considering multiple recurrent health complaints, 19.1% experienced two or more subjective health complaints more than once a week or more often.

### Well-being, future anxiety, and multiple recurrent health complaints: predictors

Table 2 reports the descriptive analysis of sex, age, SoC, SSS, DHL, satisfaction of the financial situation, presence of chronic diseases, and impairment by health problems, well-being, future anxiety, and multiple health complaints. Age distribution was not significantly associated with any health outcomes. SoC score was significantly higher with increasing well-being (median values: from 2.67 for “very low well- being” to 4.11 for “high well-being”), decreasing future anxiety (median values: from 4.22 for FA scores lower than 3.67 to 3.22 for FA scores higher than 3.67), and for students with less than two recurrent health complaints (median values: from 3.89 for those reported less than two recurrent health complaints to 2.89 for those with two or more recurrent health complaints). The distribution of DHLI score showed similar results: significantly higher scores could be found with increasing well-being (median values: from 2.83 for “very low well- being” to 2.92 for “high well-being”), decreasing future anxiety (median values: from 2.92 for FA scores lower than 3.67 to 2.83 for FA scores higher than 3.67), and for students with less than two recurrent health complaints (median values: from 2.92 for those reported less than two recurrent health complaints to 2.78 for those with two or more recurrent health complaints). Considering the categorical variables (Table 2), females were identified to have a lower mental health (lower well-being, higher FA, more frequently multiple recurrent health complaints) compared with male respondents. The same could be found for those with lower socioeconomic condition (all indicators), and for those reporting a chronic disease and accompanied impairments.

**Table 2:** Descriptive analysis of sex, SSS, satisfaction with current financial situation, presence of chronic diseases and impairment by health problems, by well-being, future anxiety, and multiple recurrent health complaints, and descriptive analysis of age, SoC and DHLI scores by well-being, future anxiety, and health complaints.

Variables		WHO-5 *N* (%)			Future anxiety scale *N* (%)		Multiple recurrent health complaints *N* (%)	
		Very low well-being	Low well-being	High well-being	<3.67	≥3.67	<2	≥2
Sex	Males (*N* = 959)	101 (10.5%)	209 (21.8%)	649 (67.7%)	572 (59.6%)	387 (40.4%)	844 (88%)	115 (12%)
	Females (*N* = 2037)	338 (16.6%)	526 (25.8%)	1,173 (57.6%)	806 (39.6%)	1,231 (60.4%)	1,581 (77.6%)	456 (22.4%)
	Diverse (*N* = 5)	2 (40%)	0	3 (60%)	2 (40%)	3 (60%)	3 (60%)	2 (40%)
	p*	<0.001			<0.001		<0.001	
SSS	Low (*N* = 408)	107 (26.2%)	123 (30.1%)	178 (43.6%)	119 (29.2%)	289 (70.8%)	288 (78.6%)	120 (29.4%)
	Medium (*N* = 2072)	283 (13.7%)	513 (24.8%)	1,276 (61.6%)	962 (46.4%)	1,110 (53.6%)	1,699 (82%)	373 (18%)
	High (*N* = 521)	51 (9.8%)	99 (19%)	371 (71.2%)	299 (57.4%)	222 (42.6%)	441 (84.6%)	80 (15.4%)
	p*	<0.001			<0.001		<0.001	
Satisfaction with financial situation	Not sufficient (*N* = 181)	38 (21%)	57 (31.5%)	86 (47.5%)	52 (28.7%)	129 (71.3%)	127 (70.2%)	54 (29.8%)
	Less sufficient (*N* = 730)	125 (17.1%)	219 (30%)	386 (52.9%)	284 (38.9%)	446 (61.1%)	553 (75.8%)	177 (24.2%)
	Sufficient (*N* = 1,523)	204 (13.4%)	364 (23.9%)	955 (62.7%)	722 (47.4%)	801 (52.6%)	1,270 (83.4%)	253 (16.6%)
	Completely sufficient (*N* = 567)	74 (13.1%)	95 (16.8%)	398 (70.2%)	322 (56.8%)	245 (43.2%)	478 (84.3%)	80 (15.7%)
	p*	<0.001			<0.001		<0.001	
Chronic disease	No (*N* = 2,561)	359 (14.0%)	607 (23.7%)	1,595 (62.3%)	1,185 (46.3%)	1,376 (53.7%)	2,105 (82.2%)	456 (17.8%)
	Yes (*N* = 440)	82 (18.6%)	128 (29.1%)	230 (52.3%)	195 (44.3%)	245 (55.7%)	323 (73.4%)	117 (26.6%)
	p*	<0.001			0.240		<0.001	
Impairment by health problems	No (*N* = 2,739)	379 (13.8%)	656 (24.0%)	1704 (62.2%)	1,279 (46.7%)	1,460 (53.3%)	2,251 (82.2%)	488 (17.8%)
	Yes (*N* = 262)	62 (23.7%)	79 (30.2%)	121 (46.2%)	101 (38.5%)	161 (61.5%)	177 (67.6%)	85 (32.4%)
	p*	<0.001			0.007		<0.001	
**Variables**		**WHO-5**			**Future anxiety scale**		**Multiple recurrent health complaints**	
		**Very low well-being**	**Low well-being**	**High well-being**	**<3.67**	**≥3.67**	**<2**	**≥2**
Age	Mean ± SD	23.6 ± 5.0	23.2 ± 4.9	23.5 ± 6.0	23.9 ± 6.6	23.1 ± 4.5	23.5 ± 5.7	23.1 ± 4.9
	Median (IQR)	22 (20–25)	22 (20–24)	22 (20–24)	22 (20–25)	22 (20–24)	22 (20–24)	22 (20–25)
	p*	0.211			0.668		<0.001	
SoC score	Mean ± SD	2.75 ± 0.97	3.20 ± 0.92	4.06 ± 0.90	4.14 ± 0.92	3.24 ± 0.99	3.82 ± 0.98	2.96 ± 1.07
	Median (IQR)	2.67 (2.0–3.44)	3.22 (2.55–3.89)	4.11 (3.44–4.67)	4.22 (3.55–4.78)	3.22 (2.55–3.89)	3.89 (3.11–4.55)	2.89 (2.22–3.67)
	p*	<0.001			<0.001		<0.001	
DHLI score	Mean ± SD	2.85 ± 0.49	2.81 ± 0.46	2.90 ± 0.48	2.92 ± 0.49	2.83 ± 0.47	2.88 ± 0.47	2.83 ± 0.51
	Median (IQR)	2.83 (2.52–3.17)	2.78 (2.5–3.08)	2.92 (2.67–3.17)	2.92 (2.67–3.17)	2.83 (2.50–3.08)	2.92 (2.58–3.08)	2.78 (2.50–3.17)
	p*	0.121			<0.001		<0.001	

^*^Fisher exact test; ^**^Kruskal–Wallis test. SSS, subjective social status; WHO, World Health Organization; SoC, sense of coherence; DHLI, digital health literacy instrument; SD, standard deviation; IQR, interquartile range.

Finally, Table 3 reports the results of multivariate logistic analyses (models A, B, and C, one for each outcome variable). Sex was a significant predictor of all the outcome variables: female students indicated a higher likelihood of poorer mental health. On the other hand, for all the outcome variables, the likelihood of reporting a better mental health status significantly increased with increasing SoC score. Conversely, socioeconomic status, either measured using SSS or satisfaction with financial situation, was excluded from the final models for well-being and multiple health complaints as not significant associations could be found. Only high SSS maintained a significant predictor for FA (OR: 1.39 of FA score lower than 3.67 with respect to equal or higher than 3.67). Moreover, the presence of an impairment by health problems emerged as another significant predictor of low or very low well-being and for two or more recurrent health complaints at least once a week.

**Table 3:** Multivariate logistic regression analysis – first and final models.

		First model (*N* = 2,902)									Final model (*N* = 3,001)								
Predictors		A			B			C			A			B			C		
		OR	95% CI	*p*	OR	95% CI	*p*	OR	95% CI	*p*	OR	95% CI	*p*	OR	95% CI	*p*	OR	95% CI	*p*
Sex	Males	1	–	–	1	–	–	1	–	–	1	–	–	1	–	–	1	–	–
	Females	0.65	0.54; 0.79	<0.001	0.42	0.35; 0.50	<0.001	0.48	0.38; 0.61	<0.001	0.66	0.55; 0.80	<0.001	0.42	0.35; 0.49	<0.001	0.35	0.38; 0.61	<0.001
	Diverse	2.22	0.16; 31.6	0.553	0.50	0.04; 5.88	0.584	0.40	0.04; 4.53	0.450	2.31	0.16; 32.4	0.532	1.96	0.04; 6.25	0.612	2.45	0.04; 4.35	0.458
SoC		3.15	2.84; 3.49	<0.001	2.63	2.84; 3.49	<0.001	2.22	2; 2.5	<0.001	3.13	2.84; 3.44	<0.001	2.56	2.38; 2.86	<0.001	0.45	2.04; 2.44	<0.001
SSS	Low	1	–	–	1	–	–	1	–	–	Excluded			1	–	–	Excluded		
	Medium	1.22	0.94; 1.59	0.135	1.19	0.91; 1.56	0.214	1.15	0.87; 1.54	0.333				1.23	0.96; 1.61	0.101			
	High	1.30	0.92; 1.84	0.131	1.37	0.98; 1.92	0.070	1.07	0.73; 1.59	0.712				1.39	1.02; 1.92	0.037			
Satisfaction with financial situation	Not sufficient	1	-	–	1	–	–	1	–	–	Excluded			Excluded			Excluded		
	Less sufficient	0.84	0.57; 1.22	0.354	1.23	0.83; 1.85	0.297	0.94	0.63; 1.41	0.783									
	Sufficient	0.82	0.56; 1.19	0.298	1.15	0.77; 1.69	0.487	1.09	0.72; 1.61	0.702									
	Completely sufficient	0.72	0.47; 1.10	0.138	1.03	0.67; 1.59	0.908	0.81	0.51; 1.30	0.377									
DHLI score		1.07	0.89; 1.29	0.442	1.10	0.92; 1.31	0.303	0.96	0.78; 1.19	0.702	Excluded			Excluded			Excluded		
Chronic disease	No	1	–	–	1	–	–	1	–	–	Excluded			Excluded			Excluded		
	Yes	0.90	0.68; 1.18	0.456	0.99	0.73; 1.33	0.939	0.81	0.60; 1.10	0.168									
Impairment by health problems	No	1	–	–	1	–	–	1	–	–	1	–	–	1	–	–	1	–	–
	Yes	0.70	0.49; 1.00	0.048	0.42	0.35; 0.50	<0.001	0.67	0.47; 0.97	0.030	0.67	0.50; 0.91	0.009	0.42	0.35; 0.49	<0.001	0.58	0.43; 0.78	<0.001

Model A – outcome variable: WHO-5 (high well-being with respect to low or very low well-being); model B – outcome variable: future anxiety (FA score < 3.67 with respect to ≥ 3.67); outcome variable: multiple recurrent health complaints (< 2 with respect to ≥ 2). *Kruskal–Wallis test. SoC, sense of coherence; SSS, subjective social status; DHLI, digital health literacy instrument; SD, standard deviation; IQR, interquartile range; OR, odd ratio; CI, confidence interval.

## Discussion

### Main finding of this study

The COVID-19 pandemic has strongly affected – and is still affecting – people’s life. University students have been overwhelmed by both the effects of social restriction and lockdowns, as well as by the uncertainty related to the socio-economic consequences of the pandemic on the medium and long-term. In this context, our study aims at describing psychological well-being, future anxiety, and health complaints in a sample of students attending the University of Florence, between the first and the second pandemic wave of COVID-19, and to assess the role of socioeconomic condition, chronic diseases, SoC and DHL as their predictors. Considering the results, it emerged that students feel threatened by economic and political situations and disturbed by not being able to achieve their goals in their future. About 40% of the students reported a low or very low well-being, and 19.1% experienced two or more subjective health complaints more than once a week or about every day. Some variables emerged as predictors of the outcome variables: sex (females presented worse condition), SoC (the higher the SoC, the better the health condition), subjective social status (the higher the SoC, the lower the anxiety for the future), chronic diseases (better condition are reported among students who did not suffer from chronic diseases). On the contrary, DHL did not predict the outcome variables.

### What is already known on this topic

Studies conducted on previous pandemics occurred over the last 60 years - the Asian flu (1956–1957), SARS (2002–2003), H_1_N_1_ flu (2009–2010), Ebola (2013–2014) - gave useful elements to evaluate the psychological reactions resulting from these public health emergencies (40). Such reactions include maladaptive behaviors, emotional distress, and defensive responses. In fact, both during and after the pandemic period, individuals had an increased likelihood of mental health problems including insomnia, anger, fear of illness, increased health risk behaviors, such as psychotropic substance use and social isolation, onset of mental disorders, such as anxiety, depression, somatization and decreased perceived health (41). Differently from the previous ones, the COVID-19 pandemic is occurring in the digital and social media era, resulting in enormous mediatic visibility and in generating an infodemic, and this could further lead to uncertainties, distress, and difficulties in making appropriate health decisions. In this perspective, DHL is a fundamental skill during the COVID-19 pandemic, that could influence mental well-being. For these reasons, our study, as part of an international project, investigated DHL, future life perspectives, health-related outcomes in a sample of university students (42). Surprisingly, in our sample, DHL does not affect well-being, FA and HCs.

Our findings in several aspects – such as in terms of demographics, SoC and DHLI scores - are comparable to the many studies published from COVID-HL network (8, 13, 17, 18, 31, 43–48). Also considering the health-related outcomes, Florentine university students presented conditions in line with the other studies. Regarding health complaints, more than half of our students suffered from an HC at least once a week in the last 6 months, in line with Dadaczynski et al. (31). On the other hand, the reported level of well-being varies, probably due to the different waves of the pandemic during which the surveys were conducted, as well as due to the different restriction measures adopted at the national or local levels and to the to the cultural differences of each country (8, 46, 48). As far as FA score is concerned, our findings are similar to what emerged from the Australian study by Dodd et al. (8), and higher than that measured in the German sample by Dadaczynski et al. (31).

According to our results, sex and SoC emerged as consistent predictors of all the investigated outcomes, also in the multivariate analysis: while females presented worse health status, the higher the SoC, the better the health condition. SoC is a resource to cope with physical isolation and social distancing and can help students to avoid or contain healthcare problems, particularly in the mental dimension. In fact, as evidenced by some studies (49–52), subjects with high SoC experience symptoms of stress less frequently and cope with stressful situations more efficiently. The potential of SoC to promote mental health during stressful situations is supported by previous studies across different populations: university students (22), adolescents (53) disadvantaged women (54), caregivers of older adults and hospital patients (55), and older adults (21). In fact, higher SOC could enable persons to perceive stressful situation, like the first phases of the pandemic, as not too bad and manageable by utilizing available resources, thereby reducing the fear of the unknown.

The role of female sex as risk condition for depression, anxiety, and stress is consistent with the studies conducted by Dodd (8), Dadaczynski et al. (31), Debowska et al. (56), and Hou et al. (57). In particular, in the face of a greater comprehensibility shown by males about the epidemic, there is a greater FA by females (31). Moreover, males tend to have higher SoC and well-being than females (58).

As for DHL and satisfaction of current economic situation, although at the bivariate analysis significant associations were found with all the health-related outcomes, their role as predictors were lost at the multivariate analysis. Also, for chronic conditions, significant associations were not confirmed at the multivariate analysis. These results seem different with respect to those reported by other Authors. In particular, some studies have described that having sufficient financial resources could also play a role in addressing people’s anxiety during the COVID-19 pandemic (59, 60). Moreover, some studies have reported that students with high levels of DHL were those who reported low levels of anxiety about the future and less somatization symptoms (8, 19, 31, 54, 55). Considering the predictors of SoC described in other studies (8, 61) as well as the parallelism between competences, skills and abilities of health literacy with respect to the three dimensions of SoC (62) we can suppose that DHL and socioeconomic condition have to be considered mostly as predictors of SoC, instead of strong and direct predictors of students’ health. This aspect emerged also by a mediation analysis performed in a sample of older people during the current pandemic, in which SoC had a direct, negative effect on anxiety and mediated the relationships between anxiety and DHL/financial satisfaction (21). A similar connection can also be supposed for chronic conditions: for people with these characteristics, the relationships between anxiety, well-being, health complaints, SoC, DHL and economic status could be complex, multiple, and non-linear. Future studies will be useful to deepen – and eventually confirm - these relationships also among university students.

### What this study adds

This study adds new elements regarding the predictors of well-being, FA and HCs of university students during the COVID-19 pandemic. In particular, to the best of our knowledge, this is the first study conducted in Italy investigating a wide range of health-related outcomes and potential predictors, by using validated scales. Because this study was conducted within an international network, our results contribute to investigate country-specific characteristics related to the impact of the COVID-19 pandemic on university students.

### Limitation of this study

This study presents some limitations. First, the participation was voluntary, and only about 6% of the students joined it. Moreover, all the information was self-reported, so underestimation or overestimation of health conditions or health-related outcomes were possible. Finally, the cross-sectional design does not allow to assess causal relationship between potential predictors and health-related outcomes.

### Generalizability

Participation was voluntary, so the study was conducted using a convenience sample, in so limiting the generalizability of the results to the entire population of students at the University of Florence.

## Conclusion

SoC and sex resulted strong predictors of well-being, multiple recurrent health complaints and future anxiety. In this perspective, health promoting interventions devoted to enhancing SoC should be conducted, in order to improve the health status of the university students, particularly in mitigating the negative consequences on mental health caused by the many stressful factors produced by the pandemic.

## Data availability statement

The raw data supporting the conclusions of this article will be made available by the authors, without undue reservation.

## Ethics statement

This study was approved by the Ethics Committee of the University of Florence (n. 108, 2020/07/07). All procedures followed were in accordance with the ethical standards of the responsible committee on human experimentation (institutional and national) and with the Helsinki Declaration of 1975, as revised in 2000. All patients provided their written informed consent to participate in this study.

## Author contributions

OO and KD: conceptualization. OO, KD, CL, and GB: methodology. CL, GB, PB, GCa, and VV: formal analysis and investigation. CL, GCa, VV, PB, and GCi: writing – original draft preparation. CL, GCa, VL, and GB: writing – review and editing. GB: resources. GB, CL, GCa, VL, and PZ: supervision. All authors contributed to the article and approved the submitted version.

## Conflict of interest

The authors declare that the research was conducted in the absence of any commercial or financial relationships that could be construed as a potential conflict of interest.

## Publisher’s note

All claims expressed in this article are solely those of the authors and do not necessarily represent those of their affiliated organizations, or those of the publisher, the editors and the reviewers. Any product that may be evaluated in this article, or claim that may be made by its manufacturer, is not guaranteed or endorsed by the publisher.
